# Tetranitrate of Erythrol

**Published:** 1899-04-15

**Authors:** 


### Editor's Letter-Box.

[Our Correspondents are reminded that prolixity is a great bar to publication, and that brevity of style and conciseness of statement greatly facilitate early insertion.]

The Reck Heating Company writes: Regarding the article about our disinfector we beg to draw attention to an error in the temperature mentioned. You say, "As a result of experiments it was found that the temperature reached within 16 folds of blanket after 35 minutes was 200 deg. Fahr." It should have been 220 deg. Fahr.

### TETRANITRATE OF ERYTHROL.

Colonel A. Ford, H.M. Chief Inspector of Explosives, writes:—An accident, by which a chemist lost his life, happened at a tabloid factory at Dartford on December 15th, 1897. He was engaged in mixing tetranitrate of erythrol with finely powdered lactose in a mortar, when an explosion occurred. Again, at the end of 1898, an accident was caused by tetranitrate of erythrol being inadvertently thrown into a fire, and one person was injured. Tetranitrate of erythrol is possessed of explosive properties, and is highly sensitive, more so, indeed, to percussion than dynamite or guncotton. As it has lately come into some use in the place of nitroglycerine as a remedy for angina pectoris, I should be glad if you would draw special attention in your paper to the dangers attending the handling of this drug.

